# Rhizoactinobacteria Enhance Growth and Antioxidant Activity in Thai Jasmine Rice (*Oryza sativa*) KDML105 Seedlings under Salt Stress

**DOI:** 10.3390/plants12193441

**Published:** 2023-09-29

**Authors:** Kawiporn Chinachanta, Arawan Shutsrirung, Choochad Santasup, Wasu Pathom-Aree, Doan Trung Luu, Laetitia Herrmann, Didier Lesueur, Chanakan Prom-u-thai

**Affiliations:** 1 Department of Plant and Soil Sciences, Faculty of Agriculture, Chiang Mai University, Chiang Mai 50200, Thailand; kawiporn.ch@cmu.ac.th (K.C.); arawan.s@cmu.ac.th (A.S.); choochad.s@cmu.ac.th (C.S.); 2 Center of Excellent in Microbial Diversity and Sustainable Utilization, Chiang Mai University, Chiang Mai 50200, Thailand; wasu.p@cmu.ac.th; 3 Research Center of Microbial Diversity and Sustainable Utilization, Department of Biology, Faculty of Science, Chiang Mai University, Chiang Mai 50200, Thailand; 4 IPSiM, CNRS, INRAE, Institute Agro, University of Montpellier, 34060 Montpellier, France; doan.luu@cnrs.fr; 5 Alliance of Bioversity International and Centre International of Tropical Agriculture (CIAT), Asia Hub, Common Microbial Biotechnology Platform (CMBP), Hanoi 10000, Vietnam; l.herrmann@cgiar.org (L.H.); didier.lesueur@cirad.fr (D.L.); 6 School of Life and Environmental Sciences, Faculty of Science, Engineering and Built Environment, Deakin University, Melbourne, VIC 3125, Australia; 7 Centre de Coopération Internationale en Recherche Agronomique pour le Développement (CIRAD), UMR Eco&Sols, Hanoi 10000, Vietnam; 8 Eco & Sols, CIRAD, INRAE, Institut de Recherche pour le Développement (IRD), Montpellier SupAgro, Université de Montpellier (UMR), 34060 Montpellier, France; 9 Chinese Academy of Tropical Agricultural Sciences, Rubber Research Institute, Haikou 571101, China; 10 Lanna Rice Research Center, Chiang Mai University, Chiang Mai 50200, Thailand

**Keywords:** aromatic rice, climate-resilient agriculture, plant-growth-promoting actinomycetes, salinity stress mitigation, salt stress alleviation, salt-tolerant rhizobacteria, microbial bioinoculants

## Abstract

Salinity is one of the most devastating abiotic stresses hampering the growth and production of rice. Nine indole-3-acetic acid (IAA)-producing salt-tolerant plant-growth-promoting rhizobacteria (ST-PGPR) were inoculated into Thai jasmine rice (*Oryza sativa* L.) variety Khao Dawk Mali 105 (KDML105) seedlings grown under different concentrations of NaCl (0, 50, 100, and 150 mM). The ST-PGPR strains significantly promoted the growth parameters, chlorophyll content, nutrient uptake (N, P, K, Ca, and Mg), antioxidant activity, and proline accumulation in the seedlings under both normal and saline conditions compared to the respective controls. The K^+^/Na^+^ ratio of the inoculated seedlings was much higher than that of the controls, indicating greater salt tolerance. The most salt-tolerant and IAA-producing strain, *Sinomonas* sp. ORF15-23, yielded the highest values for all the parameters, particularly at 50 mM NaCl. The percentage increases in these parameters relative to the controls ranged from >90% to 306%. Therefore, *Sinomonas* sp. ORF15-23 was considered a promising ST-PGPR to be developed as a bioinoculant for enhancing the growth, salt tolerance, and aroma of KDML105 rice in salt-affected areas. Environmentally friendly technologies such as ST-PGPR bioinoculants could also support the sustainability of KDML105 geographical indication (GI) products. However, the efficiency of *Sinomonas* sp. ORF15-23 should be evaluated under field conditions for its effect on rice nutrient uptake and growth, including the 2AP level.

## 1. Introduction

Salt-affected inland soil (mainly halite (NaCl)) is one of the major constraints affecting rice production in northeastern Thailand. Affected areas include Thung Kula Rong Hai (TKR), an area famous for the unique aroma and high grain quality of the Khao Dawk Mali 105 (KDML105) rice variety known as Thai Jasmine rice (*Oryza sativa* L.). While saline soils affect approximately 50% of the country’s rice-cultivated area [1], sustainable KDML105 rice production in the TKR area faces significant challenges due to the increase in soil salinity resulting from lengthened dry seasons caused by global climate change and the use of chemical fertilizers. The negative impact of excessive salinity includes an imbalance in cellular ionic flux and excessive concentrations of Na^+^, Cl^−^, Mg^2+^, K^+^, and Ca^2+^ ions inside the plant cells, thereby creating oxidative stress through the production of reactive oxygen species (ROS) that impair photosynthesis and cellular metabolism, leading to reductions in plant growth and yield [2,3]. In rice, excess soluble salts in the soil directly affect plant-growth-promoting rhizobacteria (PGPR) and reduce yield components, including stand establishment, the numbers of panicles, tillers, and spikelets per plant, floret sterility, individual grain size, and delayed heading [4,5]. However, some groups of rhizosphere microbes, particularly salt-tolerant PGPR (ST-PGPR), can survive in high-salt environments due to their ability to cope with osmotic stress; such microbes can improve plant growth as well as plant tolerance to salinity. The protective activities of ST-PGPR are related to their ability to acquire nutrients from the soil, to produce phytohormones and osmoprotectants, and to induce systemic resistance (ISR) [6,7]. Thus, these defense mechanisms could be very helpful for plants in severely saline conditions and promote plant growth under normal and stressful environmental conditions. The literature has confirmed several identified strains in various genera, e.g., *Planococcus*, *Pseudomonas*, *Bacillus*, *Enterobacter*, and *Azotobacter*, that play significant roles in improving crop yield in wheat, rice, maize, and groundnut under salinity stress [8,9,10,11]. The use of PGPR has been reported to improve the growth of non-aromatic and aromatic rice; the latter is most preferred by consumers, and the inoculation of PGPR has markedly increased the chlorophyll content, photosynthetic capacity, and growth of rice [12]. The ST-PGPR strain TY0307 exhibited a promising ability regarding salt tolerance, proline accumulation, and the yield of rice under salt-stress conditions [6,11]. Additionally, rice rhizobacterial strains of *Pseudomonas*, *Enterobacter*, and *Acinetobacter* were reported to produce 2-acetyl-1-pyrroline (2AP), the primary aromatic compound in the rice variety Basmati-370 [13]. The application of locally isolated ST-PGPR strains could be an effective long-term and sustainable solution for rice cultivation in salt-affected soils in the current agricultural systems that must cope with the effect of climate change [14,15]. Using alternative strategies for the mitigation of salinity may not be feasible, as they may have negative impacts on the agroecosystems.

The use of ST-PGPR for plant growth and maintenance of plant homeostasis under saline conditions is gaining increased attention as a strategy for solving the problem of salt stress. In the present investigation, we hypothesized that the inoculation of ST-PGPR obtained from the KDML105 rice rhizosphere grown in the TKR region would enhance the growth, salt tolerance, and aroma intensity of the KDML105 variety. Therefore, IAA-producing ST-PGPR strains previously screened by our group [16,17] were used in the present study. Firstly, the 2AP production potential in the culture broth of the ST-PGPR strains was selected [16], and the selected strains were then used to inoculate KDML105 rice seedlings. The objective of the present study was to evaluate the effects of ST-PGPR strains on growth parameters, chlorophyll content, nutrient concentration, antioxidant capacity, and proline concentrations in the rice seedlings germinated under different levels of salinity.

## 2. Results

### 2.1. Effect of ST-PGPR Inoculation on KDML105 Rice Seedlings

The inoculation effects of nine ST-PGPR strains on KDML105 rice seedlings grown under normal (0 mM NaCl) and saline conditions (50, 100, and 150 mM NaCl) were evaluated. Uninoculated seedlings at 0, 50, 100, and 150 mM NaCl were considered as control-0 (pure control), control-50, control-100, and control-150, respectively. The term ‘controls’ was used for the uninoculated seedling treatments at each of the salinity levels. These terms for each of the respective control(s) are used throughout this paper. The results showed that the inoculation of ST-PGPR had significant positive effects on the growth parameters, chlorophyll content (SPAD units), antioxidant activity (DPPH), and proline concentration of the seedlings both under normal and saline conditions.

### 2.2. Growth Parameters

**Figure 1:**
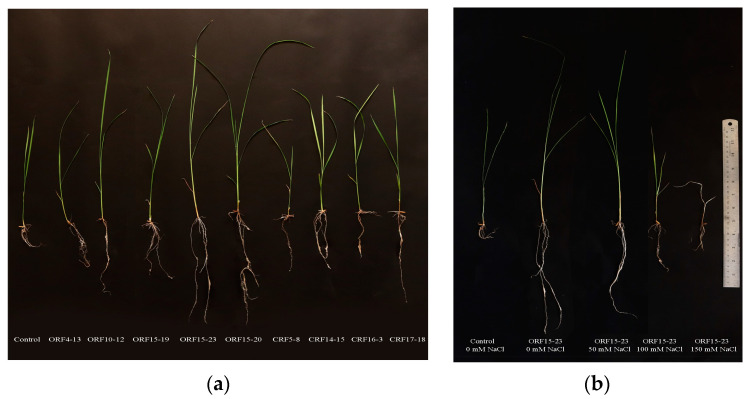
Growth-promoting effects of inoculation with ST-PGPR strains on KDML105 rice seedlings under normal condition (**a**), and examples of seedlings inoculated with *Sinomonas* sp. ORF15-23 under various NaCl concentrations (**b**).

ST-PGPR strains and salinity had significant interactions that affected both shoot and root length and dry biomass (*p* < 0.0001) (Table 1). Under normal growing condition (0 mM NaCl), the inoculation of ST-PGPR strains clearly enhanced KDML105 rice seedling growth compared to the control-0, except for strain CRF5-8. Strains *Micrococcus* sp. ORF15-20 and *Sinomonas* sp. ORF15-23 yielded the highest length for shoot and root, while CRF-5-8 showed the shortest shoot and root, similar to those of the control-0 (Figure 1a). Figure 1b shows KDML105 rice seedling growth as affected by the inoculation of ST-PGPR strain *Sinomonas* sp. ORF15-23 under various NaCl concentrations. The nine selected ST-PGPR strains differed significantly in promoting the seedling growth under salinity stress. Among all the tested strains, *Sinomonas* sp. ORF15-23, the most salt-tolerant strain, promoted the greatest shoot length and biomass at all levels of NaCl, followed by *Micrococcus* sp. ORF15-20, *Enterobacter* sp. ORF10-12, and *Micrococcus* sp. ORF15-19. In general, these growth parameters were promoted by the inoculation of most of the tested ST-PGPR strains when the NaCl concentration was increased from 0 to 50 mM, but growth declined progressively at concentrations beyond 50 mM. However, on average, the inoculated treatments provided higher seedling biomass than those of their respective controls at all levels of NaCl concentration. The non-IAA-producing strain CRF5-8 gave the lowest shoot and root length, with values slightly lower than its respective controls at each NaCl concentration. In contrast, the least salt-tolerant strain, *Burkholderia* sp. CRF16-3, provided the lowest seedling biomass, with values similar to its respective controls at each NaCl concentration.

### 2.3. Chlorophyll Content of KDML105 Rice Seedlings

Leaf chlorophyll content in all the treatments decreased gradually with the increase in salt concentration, but there were different magnitudes of decrease among the treatments. The chlorophyll contents of the control-50, control-100, and control-150 seedlings were decreased by 3.04, 7.05, and 21.04%, respectively, compared to the control-0. However, the application of ST-PGPR strains enhanced the leaf chlorophyll content of the seedlings by 1.34–18.48%, ~0–16.5%, ~0–15.2%, and ~0–27.2%, at 0, 50, 100, and 150 mM NaCl, respectively, compared to the respective controls (Table 2). The maximum chlorophyll content was obtained with *Sinomonas* sp. ORF15-23 inoculation at all levels of salinity, with the percentage of increase ranging from 15.26 to 27.50% compared to the respective controls. Under salinity stress, the inoculation of ST-PGPR strains significantly increased the total chlorophyll content compared to those of the controls.

### 2.4. Antioxidant Activity and Proline in KDML105 Rice Seedlings

It appeared that salinity had a promoting effect on the antioxidant activity (DPPH radical scavenging activity) of the leaves of KDML105 rice seedlings, and the effect was significantly enhanced by the inoculation with the ST-PGPR strains. The values of DPPH radical scavenging activity ranged from 43.94 to 60.92 mg Trolox g mL^−1^ for the uninoculated controls, and from 45.43 to 93.43 mg Trolox g mL^−1^ for treatments inoculated with ST-PGPR strains. The highest concentration of tested NaCl (150 mM) provided the maximum antioxidant activity in the seedling leaves for each treatment, with values of 60.32 to 93.43 mg Trolox g mL^−1^. On average, the antioxidant activities were ranked as *Sinomonas* sp. ORF15-23 > *Micrococcus* sp. ORF15-20 > *Micrococcus* sp. ORF15-19 > *Enterobacter* sp. ORF10-12 > *Sinomonas* sp. CRF14-15 > *Bacillus* sp. CRF17-18 > *Sinomonas* sp. ORF 4-13 > *Burkholderia* sp. CRF 16-3 > controls > CRF 5-8 (Table 2).

The proline accumulation in leaves of KDML105 seedlings increased with an increasing NaCl concentration from 0 to 100 mM NaCl and decreased thereafter. Furthermore, the inoculation with ST-PGPR strains significantly increased the proline content of the leaves at all NaCl concentrations compared to the respective controls. The maximum increase in proline content was obtained by *Sinomonas* sp. ORF15-23 inoculation, with percentage increases of 107.8, 163.0, 80.2, and 121.7% at 0, 50, 100, and 150 mM NaCl compared to control-0, control-50, control-100, and control-150, respectively (Table 2).

### 2.5. Nutrient Uptake

The relationships between NaCl concentrations, ST-PGPR inoculation, and nutrient uptake (N, P, K, Ca, Mg, and Na) of KDML105 rice seedlings are shown in Table 3. The analysis showed significant interaction (*p* < 0.01) between ST-PGPR strains and NaCl concentrations for nutrient uptake. The shoot N, P, and K uptake decreased in the controls under increasing NaCl concentration, particularly beyond 50 mM NaCl (Table 3). On average, the inoculated treatments provided higher shoot N, P, and K uptake than those of their respective controls. However, the shoot N, P, and K uptake in most of the inoculated treatments also showed a similar trend of negative salt stress effects as in their respective controls, but to a much lesser degree. Among all treatments, strain *Sinomonas* sp. ORF15-23 produced the highest levels of N, P, and K uptake at 50 mM NaCl, with percentage increases of 145.2, 186.2, and 272.6% compared to the control-50 (Table 3).

Compared to their respective controls, inoculation with most ST-PGPR strains increased shoot N, P, and K uptake in the KDML105 rice seedlings, and the highest NaCl level (150 mM) provided the greatest percentage increases at 41.6–126.2, 69.6–157.8, and 11.2–301.7%, respectively. It was interesting to note that all the CRF strains from conventional rice farming resulted in a lower N uptake than the control-50 (Table 3). Among all the tested strains, only *Sinomonas* sp. ORF15-23 clearly enhanced N, P, and K uptake when the salinity increased from 0 to 50 mM NaCl; however, the uptake decreased progressively beyond 50 mM NaCl.

Increasing salt concentration resulted in a decrease in Ca and Mg uptake by the KDML105 rice seedlings, both uninoculated and inoculated treatments, except for strain *Sinomonas* sp. ORF15-23 that showed markedly enhanced Ca and Mg uptake when the concentration increased from 0 to 50 mM NaCl (Table 4). The highest amounts of Ca and Mg uptake were obtained with the strain *Sinomonas* sp. ORF15-23 at 50 mM NaCl, with percentage increases of 306.5 and 204.9% as compared to the control-50. However, beyond this salt level, the uptake decreased monotonically. At the same level of salt concentration, the inoculation with most ST-PGPR strains increased the Ca and Mg uptake of the KDML105 rice seedlings compared to their respective controls (Table 5). On average, the highest tested NaCl level (150 mM) resulted in the maximum percentage increases in Ca and Mg uptake in the inoculated seedlings, with values of 0–184.4 and 25.0–119.4%, respectively. In contrast to Ca and Mg uptake, the Na uptake in the controls and the ORF strains from organic rice farming was slightly increased at 50 mM NaCl compared to 0 mM NaCl. Nevertheless, the uptake decreased beyond the concentration of 50 mM NaCl. The inoculation with most ST-PGPR strains increased the Na uptake of the KDML105 rice seedlings compared to their respective controls at the same levels of salt concentration (Table 4).

One of the various strategies employed by rice to survive under salt stress is maintaining a high K^+^/Na^+^ ratio in the cells. We hypothesized that the ST-PGPR inoculation might help to promote this ratio, thereby increasing the chance of survival under stress conditions. Therefore, in this experiment, we calculated the K^+^/Na^+^ ratio in the KDML105 rice seedlings to evaluate the effect of ST-PGPR inoculation. The results indicated that under normal condition (0 mM NaCl), the K^+^/Na^+^ ratio of the control-0 seedlings (1.65) and inoculated seedlings (1.33–1.80) showed similar or slightly different values (Table 5). However, the K^+^/Na^+^ ratio of the uninoculated seedlings (controls) showed a marked reduction with increasing NaCl concentrations. The K^+^/Na^+^ ratio of the ST-PGPR-inoculated seedlings was also reduced with increasing NaCl concentrations, but to a lesser degree compared to that in the controls. Among all treatments, the strain *Sinomonas* sp. ORF15-23 provided the highest K^+^/Na^+^ ratio at 50 and 100 mM NaCl (Table 5).

### 2.6. Relationships between the Study Variables by Principal Component Analysis

**Figure 2:**
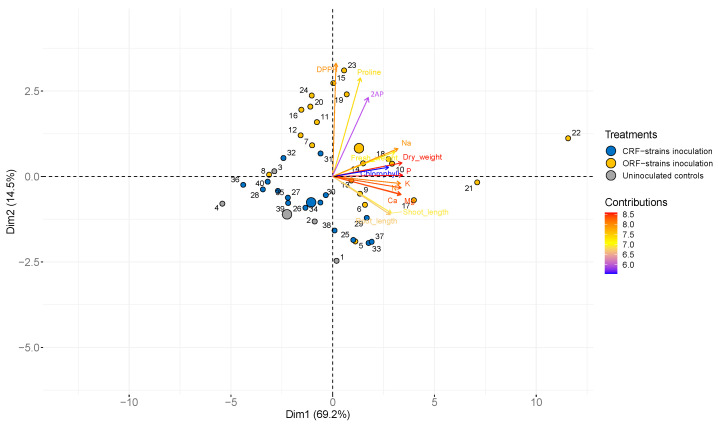
Principal component analysis showing the relationships between growth parameters (shoot and root lengths and fresh and dry biomass), chlorophyll content, nutrient (N, P, K, Ca, Mg, and Na) uptake, antioxidant activity, proline accumulation, and 2AP level of KDML105 rice seedlings as affected by ST-PGPR inoculation.

The principal component analysis (PCA) explained 83.7% of the study variables. The first principal component, PC1, explained 69.2%, and the second, PC2, explained 14.5% of the variation (Figure 2). All of the study variables were positively influenced by inoculation with the ORF strains. A close positive relationship existed between the nutrient uptake and the seedling biomass. Na showed stronger positive correlations with 2AP, proline, and DPPH (antioxidant activity) than with other nutrients. The proline level had the highest positive correlations with both antioxidant activity and 2AP level. The ORF strains had stronger positive relationships with the growth parameters, chlorophyll content, proline level, antioxidant activity, and nutrient uptake in fresh leaves of KDML105 rice seedlings than the CRF strains.

## 3. Discussion

The premium aromatic rice variety Khao Dawk Mali 105 (KDML105) comprises about 50% of the rainfed paddy rice production in a huge area of Thung Kula Rong Hai (TKR) in northeastern Thailand. In addition, the KDML105 rice produced in the TKR region possesses a stronger aroma than rice cultivated in other areas of the country, as well as in other countries [18], and thus it is traded as a premium-quality rice with a high price tag in both local and global markets. It is well known that the yield and aroma quality of KDML105 in the TKR region has been negatively affected by naturally high salinity and drought conditions [19]. The problem is exacerbated by the increase in drought as a result of global climate change. The use of salt-tolerant plant-growth-promoting rhizobacteria (ST-PGPR) is a promising, sustainable, and cost-effective alternative to chemical management that can be used to mitigate these problems [14,15]. In the present study, the shoot and root biomass, as well as the chlorophyll content (SPAD unit), of the KDML105 rice seedlings were significantly enhanced by most of the selected ST-PGPR compared to their respective controls (Table 1 and Table 2). Several other studies have confirmed that seed priming and inoculation with ST-PGPR improves rice seed germination, chlorophyll content, and photosynthetic capacity as well as rice growth and yield [11,12,20]. In the present study, it was interesting to note that the highest IAA-producing and most salt-tolerant ST-PGPR, *Sinomonas* sp. ORF15-23, also yielded the highest chlorophyll content and the highest values for other rice growth parameters at all levels of salinity (Table 2). In contrast, the inoculation with the non-IAA-producing strain CRF5-8 [17], as well as the least salt-tolerant strain, *Burkholderia* sp. CRF16-3, resulted in the lowest values for seedling growth parameters, which were similar to the respective controls (Table 1). This phenomenon highlighted the importance of the IAA-producing and salt-tolerance properties of the PGPR in promoting rice growth under salt stress. IAA has been demonstrated to increase root growth and surface area, leading to higher nutrient uptake and thereby improving plant growth as well as stress tolerance [21,22,23]. Previous studies showed that the growth-promoting effects on rice under salt stress are attributable to strain variability in ST-PGPR, which could enhance salt tolerance by altering root morphology, modifying root-to-shoot communication, increasing nutrients uptake, maintaining ion homeostasis, decreasing oxidative damage, and elevating photosynthetic capacity [24,25,26,27]. Therefore, key specific microbial species, not the microbial richness or diversity, determined the efficiency of growth promotion by each individual ST-PGPR. In the present study, ST-PGPR inoculation not only promoted rice seedling growth but also improved shoot N, P, K, Ca, and Mg uptake in the seedlings, particularly inoculation with *Sinomonas* sp. ORF15-23, compared to the uninoculated seedlings (Table 3 and Table 4). It is possible that *Sinomonas* sp. ORF15-23 was more compatible with the rice KDML105 than other ST-PGPR. However, the exact mechanisms behind this observation remain to be determined. Genomic analysis of the whole genome sequence and transcriptomics would allow us to gain insights into the growth-promoting and salt-tolerance mechanisms of *Sinomonas* sp. ORF15-23, as exemplified in recent publications [28,29]. In other words, the use of promising ST-PGPR effectively mitigated the deleterious effect of excessive salinity levels. It would be interesting to continue examining the effect of ST-PGPR inoculation on grain productivity and quality under practical field conditions in future studies.

Apart from stimulating plant growth, IAA produced by ST-PGPR also performs a key role in ameliorating stress in plants. Phytohormone-producing bacteria increase plant tolerance to salinity stress, thereby promoting plant growth under excessive salinity [8,30,31,32]. Auxin produced by *Bacillus amyloliquefaciens* RWL-1 has been reported to increase salinity stress tolerance in rice (*Oryza sativa* L.) [31]. Rangseekaew et al. [33,34] investigated three plant-growth-promoting abilities (IAA and siderophore production and phosphate solubilization). The IAA production by actinobacteria *D. abyssi* MT1.1^T^ at 150 mM NaCl was three-fold decreased as compared to the production at 0 mM NaCl. Similarly, reductions in IAA production by *D. profundi* MT2.2^T^ (decreased from 12.20 to 7.73 µg mL^−1^) and *D. nishinomiyaensis* DSM20448^T^ (decreased from 16.64 to 9.39 µg mL^−1^) were recorded at 150 mM NaCl. There is some evidence that IAA production is increased with increasing NaCl concentration. The results of our previous study indicated that the highest IAA-producing strain, *Sinomonas* sp. ORF15-23, could grow best under salt stress [17]. *Sinomonas* sp. ORF15-23 also exhibited the greatest ability to promote rice seedling growth in the present study (Table 1). Our results implied that, in addition to it having mechanisms for stress tolerance (e.g., IAA production, antioxidant activity, and potassium intake) [17], ST-PGPR also transmitted some level of tolerance to the rice seedling under green houses. Salt stress causes osmotic stress in the early phases, leading to the accumulation of reactive oxygen species (ROS) that are harmful to plant cells. For example, hydrogen peroxide (H_2_O_2_), an important nonradical ROS, was found to increase in tomato under 150 mM NaCl stress compared to non-inoculated tomato without salt stress [33,34]. Antioxidant activity plays a vital role in detoxifying ROS induced by salinity stress [35]. In the present study, the antioxidant activity (DPPH radical scavenging activity) in the leaves of the rice seedlings increased with an increasing salt concentration, and the activity was significantly enhanced by the inoculation with ST-PGPR strains (Table 2).

To maintain osmotic balance and optimum ROS concentration under stress conditions, plants synthesize antioxidants and osmoprotectants (osmolytes) such as proline [33,34,36,37], an amino acid that is one of the most important osmolytes in response to salinity stress. In the present study, the proline content significantly increased with the inoculation of ST-PGPR strains, particularly *Sinomonas* sp. ORF15-23, which provided the maximum proline increase (163%) at 50 mM NaCl (Table 2). In addition, the PCA indicated a close relationship between DPPH radical scavenging activity and proline level (Figure 2). Proline accumulation in plants is a primary defense response to environmental stresses, including excessive salinity. The role of proline during stress generally includes osmotic adjustment, detoxification of ROS, and protection of membrane integrity, as well as storage of organic carbon and nitrogen [38,39]. Under stressful conditions, it has been observed that proline also functions as a radical scavenger, thus performing a dual function as an osmolyte compound and an antioxidant [40]. Several studies have shown that proline effectively enhanced the salt tolerance and growth of various crops such as olives, tobacco, and rice seedlings [41,42,43]. The inoculation of bacterial isolate RWL-1 yielded greater synthesis of various amino acids, including proline, under salinity stress [31]. Under salt-stress conditions, proline accumulation was observed in rice inoculated with ST-PGPR strain TY0307, resulting in enhanced salt tolerance, growth, and yield of rice [11]. Soil salinity induces adverse effects on seedling establishment and plant biomass accumulation [44,45]. Although rice possess inherent salt-tolerant strategies (4 dS m^−1^), excessive soil salinity can damage seedling establishment and further inhibit the growth of rice and soil microbes that are of pivotal importance for plant growth, especially in adverse ecosystems such as those with saline soil conditions [46]. If the intensity increases in growing conditions, it will affect the number of microorganisms, which will decrease and reduce activities that are beneficial to plants. Our results confirmed the increase in proline accumulation in rice when exposed to salinity stress and the enhancement of proline production by ST-PGPR inoculation that enhanced salt tolerance in the rice seedlings and thereby improved the growth of seedlings during salt stress (Table 2). In addition to its function as an osmoprotectant and an antioxidant, proline has been recognized as the key precursor for the biosynthesis of 2AP, a major volatile compound of aromatic rice, including the KDML105 variety [47,48]. Several investigations have concluded that the 2AP content of KDML105 rice seedlings was markedly enhanced when exposed to salt stress, and this can be attributed to an increased accumulation of its precursor proline [47,49,50,51]. Our findings agreed with these previous studies in that the 2AP content of all the treatments increased along with the proline content in KDML105 rice seedlings under salt stress, particularly between 0 and 50 mM NaCl (Table 2). The PCA indicated that the proline level (Figure 2) had the highest positive correlations with both 2AP and antioxidant activity [17,52]. In addition, the results of this study indicated that the 2AP level was significantly higher in the inoculated seedlings than in the uninoculated seedlings. It is interesting to note that the high 2AP-producing ST-PGPR strains *Sinomonas* sp. ORF15-23, *Enterobacter* sp. ORF10-12, and *Burkholderia* sp. CRF16-3 yielded the maximum 2AP content in the seedlings at 50, 100, and 150 mM NaCl, respectively (Table 6). A previous study has shown that inoculation with high 2AP-producing rhizobacterial strains could increase the 2AP levels in the grains of the aromatic rice variety Basmati-370 [13].

In addition to osmoregulation and ROS scavenging (antioxidant activity), ion homeostasis (acid–base balance) is also considered an important defense mechanism of rice against salinity stress. The main toxic salt ions damaging to crop plants are Na^+^ and Cl^−^ [53]. Under salt stress, extracellular Na^+^ inhibits root K^+^ uptake; therefore, a high K^+^/Na^+^ ratio is important for salt tolerance. In the present study, the ST-PGPR-inoculated seedlings had a higher K^+^/Na^+^ ratio than the uninoculated seedlings, and this may have led to the higher salt tolerance (Table 5). The inoculation of *Azospirillum* to salt-stressed maize restricted Na^+^ uptake and enhanced the uptake of K^+^ and Ca^2+^ in cv. 323, thus maintaining a high K^+^/Na^+^ ratio. The K^+^/Na^+^ ratio was significantly higher in the salt-tolerant maize cv. 324 than in the salt-sensitive cv. 323 [54]. Under stressful conditions, IAA was shown to increase both proline and K contents and improve the nutritional, physiological, and metabolic activities of the plant [55]. Our observations are in accordance with this previous report in that the inoculation with the highest IAA-producing strain, *Sinomonas* sp. ORF15-23, resulted in the highest proline, Ca, and K uptake under salt stress (Table 2, Table 3 and Table 4). The increase in proline, Ca, and K uptake might have led to improvements in the growth and salt tolerance of the rice seedlings. Therefore, K is one of the vital nutrients playing a critical role in plant stress. It has been observed that high-affinity Na^+^ uptake was found in K^+^-starved seedlings of several cereal crops, including rice. Furthermore, the Na^+^ uptake was very rapid, and the Km value was low under low K^+^ and Ca^2+^ concentrations. However, high-affinity Na^+^ uptake was sensitive to external K^+^ [56,57]. These previous findings emphasize the importance of K in enhancing rice growth and salt tolerance under high salinity; thus, K should be available in sufficient quantity, particularly in the rhizosphere soil, throughout the growing season. One possible explanation could be that the exudation of specific compounds from ST-PGPR, and the growth promotion of roots, both contributed to the stimulation of microbial activity and modified the nutritional status in the rhizosphere under salt stress conditions [58,59,60,61]. The enhanced activities of IAA production, antioxidant activity, and potassium intake by ST-PGPR could benefit the transformation of soil nutrients (such as K^+^ and Na^+^) and further promote the overall availability of soil nutrients.

The results of this study revealed the promising benefits of the ST-PGPR strains for rice growth and aromatic quality (2AP) under both normal and saline conditions. The PCA indicated that the ST-PGPR rhizobacteria from organic rice farming practice (ORF strains) had stronger positive relationships with each of the study variables than those from conventional rice farming practice (CRF strains) (Figure 2). Several studies have shown that plant adaptation to local/stress environments is driven by the co-adaptation of plants and rhizosphere microbes via a complex hormonal signaling pathway [62,63], and IAA appears to play a major role in microbe–plant interactions [64]. Exposure to excessive salinity was found to decrease maize and wheat root attachment by *Azospirillum brasilense* [65]. Similar findings were observed in this study, as seen in the decrease in rhizobacterial count with increasing salinity. However, the highest IAA-producing strain, *Sinomonas* sp. ORF15-23, maintained the highest count at 108 CFU mL^−1^ under all NaCl levels (Table 6). The high number of *Sinomonas* sp. ORF15-23 may be the reason for its ability to promote rice seedling growth and salt tolerance. Therefore, the use of ST-PGPR(s) could be an alternative option for alleviating salinity problems and enhancing rice yield and quality in KDML105 rice grown in inland salt-affected areas such as Thung Kula Rong Hai (TKR). However, the use of the ST-PGPR inoculants in actual field conditions requires further investigation.

## 4. Materials and Methods

### 4.1. Rice Rhizobacterial Isolates

Nine KDML105 rice rhizobacterial strains that exhibited various degrees of tolerance to high salt concentrations (0 to 3% NaCl) were selected from our previous study [16] to evaluate their effects on KDML105 rice seedlings’ growth and salt tolerance. All of the strains were able to produce IAA and promote the production of 2AP in KDML105 rice seedlings under salt stress. These selected strains were considered as salt-tolerant plant-growth-promoting rhizobacteria (ST-PGPR). Five and four isolates were obtained from organic rice farming (ORF) and conventional rice farming (CRF), respectively [66]. *Micrococcus* sp. ORF15-19 and *Sinomonas* sp. ORF15-23 displayed the highest levels of salt tolerance, while *Burkholderia* sp. CRF16-3 displayed the lowest salt tolerance (Table 6).

### 4.2. Effect of ST-PGPR Inoculation on Rice Seedling Growth under Salt Stress

The ability of the nine selected ST-PGPR strains in enhancing KDML105 rice seedling growth under various NaCl concentrations was determined. The responses of the seedlings to ST-PGPR inoculation were evaluated by analysis of the following: growth parameters, chlorophyll content, nutrient concentration, antioxidant activity, and proline accumulation.

#### Preparation of ST-PGPR Pellets and Rice Seedlings

This experiment was conducted using a completely randomized design (CRD) in a factorial scheme (10 × 4), with three replications, consisting of nine selected ST-PGPR strains plus one uninoculated control (ten treatments) and four NaCl concentrations (0, 50, 100, and 150 mM NaCl).

The nine selected ST-PGPR strains were grown in 25 mL nutrient broth (NB) for three days at 37 °C with shaking at 120 rpm. The ST-PGPR cells were collected by centrifugation at 10,000 rpm for 15 min to separate the culture broth from the pellet cells. The cell pellets were diluted with 100 mL sterile distilled water to obtain a cell concentration of 10^6^ colony-forming units (CFU) per mL (OD_600_~0.2). This cell suspension was used as inoculum for seed biopriming and seedling inoculation. Sterile distilled water was used as the negative control (without ST-PGPR inoculation).

Rice (*Oryza sativa* L.) seeds variety KDML105 were used to evaluate the ability of selected ST-PGPR to promote growth and salt tolerance in rice. The seeds were surface sterilized in a mixture of 0.2% Tween 80 and 2% sodium hypochlorite for 3 min. The seeds were then washed three times with 70% ethanol, followed by rinsing five times with sterile water. The sterile seeds were soaked (seed biopriming) in the pellet suspension of each ST-PGPR strain according to the treatment and were then incubated in the dark at 25 °C for 24 h [67]. The bioprimed seeds were then placed at an equal distance on sterile wet tissue paper in a Petri dish (20 seeds per plate) using sterile forceps (five replicates per treatment) and kept in a plant growth chamber under the dark at 25 °C. Four days after germination, 10 uniform seedlings from each treatment were selected and transplanted into a growth pouch containing Hoagland’s nutrient solution (pH 7). The rice seedlings were initially irrigated with 1⁄4 strength Hoagland solution for five days, and the solution was replaced twice during this period. Then, the seedlings were irrigated with 1⁄2 strength Hoagland solution for two days. After that, the irrigation medium was changed to a full-strength Hoagland solution [68] with four salinity levels (0, 50, 100, and 150 mM NaCl). The average EC of the irrigation medium at each NaCl concentration was 2.06, 7.69, 13.78, and 19.51 dS m^−1^, respectively. The full-strength solution was refreshed twice per week. The uninoculated (controls) and inoculated seedlings were grown in a climate-controlled room (12:12 light: dark photoperiod, 25 ± 3 °C, with a light level of approximately 5.8 klux).

The rice seedlings from each pouch were harvested at 30 days after transplanting, and then four replications of the seedlings were determined for growth parameters (shoot and root length; shoot and root dry weight). The leaves and root samples were dried to a constant weight at 65 °C for 48 h. After that, the dry matter was weighed, and the dried samples were milled into powder, stored in plastic bags, and then kept in a desiccator for analysis of nutrient content. The remaining fresh seedlings (six replications) were used to determine antioxidant activity and proline accumulation in the leaves.

### 4.3. Chemical Analysis

Leaf chlorophyll content was monitored at the third leaf stage after applying the salt stress to the seedlings (at 30 days) using a SPAD meter (SPAD-502, Minolta Camera Co., Ltd., Osaka, Japan). The dried plant leaves were ground, homogenized, and used to determine the concentration of macronutrients. The total nitrogen (N) content (%) was determined by using a modified Kjeldajl digestion (colorimetric) method [69]. The digestion was maintained at a boiling point of 350 °C. Ammonia was distilled from an alkaline medium and absorbed in an unstandardized boric acid solution and titrated with standard HCl solution. For the determination of total phosphorus (P), potassium (K), calcium (Ca), magnesium (Mg), and sodium (Na), the method described by Fageria [70] was applied. The total P concentration (%) in the samples was quantified spectrophotometrically using the vanado-molybdate phosphoric acid yellow color method [71], with a UV-visible spectrophotometer (Shimadzu UV-VIS 1201, Shimadzu Co. Kyoto, Japan). The concentrations of K, Ca, Mg, and Na in the sample extracts were analyzed by an atomic absorption spectrophotometer (AAS) (Spectra AA240 FS, Varian, California, USA). Each sample was measured in triplicate. The nutrient uptake was calculated from the nutrient concentration and the dry matter of each sample using the following formula.

(1) $$\begin{array}{c} Nutrient\;uptake(mg\;{plant}^{-1})=\frac{Nutrient\;content\left(\%\right)\times Dry\;matter\left(mg\;{plant}^{-1}\right)}{100}\\ \mathrm{Nutrient}\;\mathrm{uptake}\;=\;g\;{\mathrm{plant}}^{-1}\;\left(\mathrm{macronutrients}\right)\;\mathrm{or}\;\mathrm{mg}\;{\mathrm{plant}}^{-1}\;\left(\mathrm{micronutrients}\right)\\ \mathrm{Nutrient}\;\mathrm{content}\;\left(\%\right)\;=\;\mathrm{Element}\;\mathrm{concentration}\;=\;\mathrm{in}\;g\;{\mathrm{kg}}^{-1}\;\left(\mathrm{for}\;\mathrm{macronutrients}\right)\;\mathrm{or}\\ \mathrm{mg}\;{\mathrm{kg}}^{-1}\;\left(\mathrm{for}\;\mathrm{micronutrients}\right)\\ \mathrm{Dry}\;\mathrm{matter}\;=\;\mathrm{shoot}\;\mathrm{dry}\;\mathrm{weight}\;=\;\mathrm{in}\;g\;{\mathrm{plant}}^{-1}\;\left(\mathrm{for}\mathrm{macronutrients}\right)\;\mathrm{or}\;\mathrm{mg}\;{\mathrm{kg}}^{-1}\\ \left(\mathrm{for}\;\mathrm{micronutrients}\right) \end{array}$$

For the antioxidant activity analysis, the oven-dried leaf samples were defatted twice with hexane (1:20 *w*/*v*) for 30 min. The defatted rice leaf fraction was extracted twice with 99.9% methanol (1:20 *w*/*v*) in an electrical shaker overnight at room temperature and then filtered through Whatman No.1 filter paper. The extracts were evaporated to dryness at 50 °C by a vacuum rotary evaporator. The extract in the evaporator flask was eluted with methanol to a volume of 100 mL, then kept in a volumetric flask. The extracts were stored in the freezer at −18 °C until use in further analysis. All analyses were performed within two weeks of extraction.

The free radical scavenging capacity was estimated following a previously reported procedure using 2,2′-diphenyl-1-picrylhydrazyl radical (DPPH) [72]. A synthetic antioxidant, BHT (99.0% purity, Rankem, India), was used as a reference. DPPH free radical-scavenging ability was calculated using the following formula:

(2) $$\begin{array}{ll} \mathrm{Scavenging}\;\mathrm{ability}\;(\%)\;= & [\mathrm{Absorbance}\;\mathrm{at}\;517\;\mathrm{nm}\;\mathrm{of}\;\mathrm{the}\;\mathrm{control}\;-\;\mathrm{Absorbance}\mathrm{at}\;517\;\mathrm{nm}\\ & \mathrm{of}\;\mathrm{the}\;\mathrm{sample}]/\mathrm{Absorbance}\mathrm{at}\;517\;\mathrm{of}\;\mathrm{the}\;\mathrm{control}\;\times \;100. \end{array}$$

Proline content was determined by standard method as described by [33]. Dried leaf powder of each sample (0.1 g) was used to extract the proline and the absorbance of the leaf extract was measured at 520 nm by a spectrophotometer (Shimadzu UV-VIS 1201, Shimadzu Co., Kyoto, Japan), and it was recorded against pure toluene as a reference blank. The proline concentration was calculated from a standard curve prepared from pure proline (Sigma).

### 4.4. Statistical Analysis

Two-way ANOVA together with LSD values at a 1% probability level [73] was used for analyzing collected data using Statistix 9 (Analytical Software, Inc., Tallahassee, FL, USA). Principal component analysis (PCA) is a statistical technique that allows easier analysis of a large dataset with visualization by reducing the complexity and noise of the data, and highlighting the most important features and relationships between observed parameters. In this study, the relationships between the growth parameters (shoot and root length and fresh and dry biomass), chlorophyll content, nutrient uptake (N, P, K, Ca, Mg, and Na), antioxidant activity, proline, and 2AP level accumulation of KDML105 rice seedlings as affected by ST-PGPR inoculation were evaluated using PCA. The measured parameters were introduced as variables in the PCA using R 1.2.1335 [74].

## 5. Conclusions

The present investigation revealed that inoculation with most of the tested ST-PGPR strains, particularly *Sinomonas* sp. ORF15-23, significantly reduced the extent of growth suppression due to excessive salinity, leading to incremental increases in rice seedling growth and salt tolerance. In addition, the 2AP (a key volatile aroma compound) level in the rice seedlings was markedly enhanced by ST-PGPR inoculation, and this may have led to high 2AP levels in the rice grains. These findings suggest that *Sinomonas* sp. ORF15-23 can be used to enhance KDML105 rice seedling growth and improve soil nutrient uptake in saline soil. This information provides a basis for the development of a microbial technology to aid in the restoration of saline-degraded areas. Nevertheless, further investigations under field conditions are needed for the development of the promising ST-PGPR strain(s) as a bio-inoculant for rice production in salinity-affected areas, such as studies of the effects of ST-PGPR inoculation on grain quality and yield.

## Figures and Tables

**Table 1:** Growth-promoting effects of ST-PGPR inoculation on shoot length, root length, and dry weight of KDML105 rice seedlings under normal and saline conditions.

ST-PGPR Strains	Shoot Length (cm)				Root Length (cm)				Dry Weight (g)			
	NaCl Concentrations (mM)				NaCl Concentrations (mM)				NaCl Concentrations (mM)			
	0	50	100	150	0	50	100	150	0	50	100	150
**Controls ^1^**	21.72 d	20.23 d	10.60 d	5.43 d	9.59 cd	10.18 c	5.33 b	3.12 c	0.30 b	0.27 b	0.24 ab	0.16 c
**Organic rice farming**												
ORF4-13	24.41 bc	23.74 cd	18.25 ab	12.84 a	19.30 a	12.66 bc	5.67 b	3.43 c	0.32 b	0.38 b	0.29 ab	0.25 bc
ORF10-12	25.17 bc	29.51 abc	17.44 abc	14.96 a	10.01 cd	15.26 b	11.11 a	9.10 a	0.32 b	0.4 b	0.28 ab	0.28 ab
ORF15-19	23.48 cd	28.96 abc	9.69 d	7.59 b	9.83 cd	12.78 bc	4.89 b	3.77 c	0.31 b	0.34 b	0.34 a	0.29 ab
ORF15-20	27.42 ab	30.78 ab	15.80 bc	13.24 a	22.03 a	12.80 bc	6.36 b	5.14 bc	0.42 ab	0.39 ab	0.36 a	0.35 a
ORF15-23	29.42 a	32.50 a	18.81 a	15.40 a	19.34 a	20.77 a	11.48 a	6.88 ab	0.54 a	0.58 a	0.32 a	0.27 ab
**Conventional rice farming**												
CRF 5-8	20.84 d	13.43 e	9.18 d	8.23 b	7.78 d	6.83 c	4.89 b	3.87 c	0.35 b	0.26 b	0.27 ab	0.25 bc
CRF14-15	23.84 cd	24.50 cd	15.84 bc	6.84 b	11.36 bc	12.83 bc	10.55 a	4.09 c	0.32 b	0.27 b	0.30 ab	0.28 ab
CRF16-3	26.65 abc	22.37 d	19.14 a	7.35 b	10.69 cd	11.36 c	11.44 a	3.65	0.36 b	0.28 b	0.22 b	0.20 bc
CRF17-18	25.04 bc	25.00 bcd	15.00 c	7.35 b	14.38 b	13.24 bc	11.62 a	5.25 bc	0.36 b	0.31 b	0.27 ab	0.25 bc
Mean	25.10	25.102	14.98	9.92	13.43	12.87	8.33	4.82	0.36	0.37	0.29	0.26
F-test	*	*	*	*	*	*	*	*	*	*	*	*
% CV	5.76	10.00	8.31	14.34	10.39	11.10	15.48	8.04	7.64	6.90	8.25	5.11
Rhizobacterial isolates (A)	**				**				**			
NaCl concentration (B)	**				**				**			
A × B	**				**				**			
LSD_(0.01)_ (A × B)	2.61				2.08				0.08			
% CV	8.56				13.15				17.15			

^1^ Controls = control-0, control-50, control-100, and control-150 at 0, 50, 100, and 150 mM NaCl, respectively; Mean (*n* = 3). The average values followed by different letters within the same column were significantly different according to pairwise comparisons using an LSD test (*p* ≤ 0.01). *, ** Significant at the 0.05 and 0.01 probability level, respectively.

**Table 2:** Effect of ST-PGPR inoculation on chlorophyll content, proline accumulation, and antioxidant activity in the KDML105 rice seedling leaves under different NaCl concentrations.

ST-PGPR Strains	Chlorophyll (SPAD Unit)				Proline				DPPH Radical Scavenging Activity			
					(μmolg^−1^ FW min^−1^)				(mg Trolox g mL^−1^)			
	NaCl concentrations (mM)				NaCl Concentrations (mM)				NaCl Concentrations (mM)			
	0	50	100	150	0	50	100	150	0	50	100	150
**Controls^1^**	37.33 c	36.23 d	34.87 bc	30.84 c	14.84 d	17.84 f	30.43 d	15.94 d	43.94 f	57.47 cd	61.92 e	60.92 d
**Organic rice farming**												
ORF4-13	39.84 bc	36.24 d	34.95 bc	30.36 c	25.04 b	29.93 cd	38.03 c	21.93 c	48.95 df	56.04 de	68.93 cd	70.32 c
ORF10-12	40.32 bc	39.84 abc	38.74 a	37.72 a	29.05 a	35.29 b	42.93 b	29.84 b	62.04 b	71.52 b	76.34 b	80.43 b
ORF15-19	41.52 ab	40.95 ab	39.95 a	38.87 a	26.94 b	31.94 c	45.92 b	28.92 b	70.32 a	74.06 ab	83.94 a	89.32 a
ORF15-20	39.42 bc	39.42 abcd	35.39 b	33.28 b	24.95 bc	32.94 bc	45.23 b	30.94 b	69.94 a	74.95 a	82.95 a	89.42 a
ORF15-23	44.23 a	42.19 a	40.19 a	39.32 a	30.84 a	46.92 a	54.83 a	35.34 a	72.94 a	76.04 a	85.03 a	93.43 a
**Conventional rice farming**												
CRF5-8	37.93 c	36.92 cd	31.94 cd	30.48 c	16.94 c	24.92 e	23.02 e	20.94 c	45.43 ef	49.54 f	54.03 f	60.32 d
CRF14-15	40.32 bc	38.84 bcd	34.96 bc	32.05 bc	23.94 bc	27.94 d	34.29 cd	20.94 c	56.93 c	60.30 c	69.83 c	73.95 bc
CRF16-3	37.83 c	36.93 cd	33.64 bcd	30.59 c	15.94 d	28.94 cd	27.43 de	16.92 d	52.43 d	56.03 de	65.47 de	69.34 c
CRF17-18	39.82 bc	38.85 bcd	30.50 d	31.93 bc	17.94 c	18.42 f	20.58 f	15.93 d	49.95 d	53.05 e	62.05 e	70.93 c
Mean	39.86	38.64	35.51	33.54	32.52	36.51	39.78	30.22	57.29	62.90	71.05	75.84
F-test	*	*	*	*	*	*	*	*	*	*	*	*
% CV	3.37	3.62	3.86	2.94	4.31	5.47	4.42	3.72	2.95	2.13	2.18	3.86
Rhizobacterial isolates (A)	**				**				**			
NaCl concentration (B)	**				**				**			
A × B	**				**				**			
LSD_(0.01)_ for (A × B)	1.99				3.16				1.80			
%CV	3.33				2.91				3.34			

^1^ Controls = control-0, control-50, control-100, and control-150 at 0, 50, 100, and 150 mM NaCl, respectively; Mean (*n* = 3). The average values followed by different letters within the same column were significantly different according to pairwise comparisons using an LSD test (*p* ≤ 0.01). *, ** Significant at the 0.05 and 0.01 probability level, respectively.

**Table 3:** Effect of ST-PGPR inoculation on nitrogen (N), phosphorus (P), and potassium (K) uptake by KDML105 rice seedlings under different NaCl concentrations, and significance level and LSD values for nutrient uptake by KDML105 rice seedlings.

ST-PGPR Strains	N				P				K			
	(mg N plant^−1^)				(mg P plant^−1^)				(mg K plant^−1^)			
	NaCl Concentrations (mM)				NaCl Concentrations (mM)				NaCl Concentrations (mM)			
	0	50	100	150	0	50	100	150	0	50	100	150
**Controls ^1^**	10.86 d (0.00)	10.26 de (0.00)	6.264 d (0.00)	3.74 d (0.00)	3.63 b (0.00)	3.21 bc (0.00)	2.69 bc (0.00)	1.34 c (0.00)	16.41 c (0.00)	10.26 f (0.00)	8.04 e (0.00)	3.92 c (0.00)
**Organic rice farming**												
ORF4-13	11.26 d (3.72)	10.03 def (−2.22)	9.19 bc (46.76)	8.48 a (126.36)	3.90 ab (7.55)	4.52 bc (40.74)	3.36 abc (25.15)	2.73 ab (102.75)	17.09 c (4.13)	18.09 b (76.30)	11.11 cd (38.15)	9.58 b (144.26)
ORF10-12	11.58 d (6.67)	15.48 b (50.88)	9.58 bc (52.87)	6.80 abc (81.73)	3.87 ab (6.67)	4.76 b (48.15)	2.66 bc (−1.04)	2.44 abc (81.25)	17.34 c (5.67)	14.76 bcd (43.86)	9.52 de (18.41)	10.84 b (176.43)
ORF15-19	10.63 d (−2.09)	11.22 cd (9.36)	11.66 a (86.17)	7.92 ab (111.46)	3.75 b (3.33)	3.91 bc (21.69)	3.67 ab (36.61)	2.96 ab (120.09)	16.83 c (2.58)	18.22 b (77.62)	16.63 a (106.79)	15.75 a (301.71)
ORF15-20	19.45 a (79.06)	13.77 bc (34.18)	9.32 bc (48.85)	6.27 abc (67.33)	5.17 ab (42.31)	4.68 bc (45.66)	4.07 a (51.34)	3.47 a (157.81)	17.93 c (9.29)	16.30 bc (58.89)	14.80 ab (84.03)	9.03 b (130.36)
ORF15-23	17.17 ab (58.12)	25.16 a (145.19)	10.02 ab (59.90)	6.94 abc (85.34)	6.59 a (81.49)	9.20 a (186.21)	3.65 ab (35.71)	2.97 ab (116.96)	28.24 a (72.10)	38.23 a (272.59)	12.90 bc (60.40)	9.48 b (127.30)
**Conventional rice farming**												
CRF5-8	11.83 d (8.93)	8.58 def (−16.37)	8.15 c (30.17)	5.30 cd (41.56)	3.57 b (−1.65)	2.96 c (−7.75)	3.35 abc (24.55)	2.63 ab (95.31)	18.06 bc (10.05)	12.45 def (21.38)	12.39 c (54.14)	8.91 b (141.71)
CRF14-15	10.72 d (−1.29)	8.15 ef (−20.53)	10.41 ab (66.19)	8.12 a (116.88)	3.94 ab (8.43)	3.24 bc (0.84)	3.36 abc (25.00)	3.19 ab (131.25)	18.53 bc (12.91)	13.85 cde (35.00)	12.69 bc (57.84)	11.12 ab (183.67)
CRF16-3	15.08 bc (38.90)	8.85 def (−13.76)	5.85 d (−6.58)	5.78 bcd (54.38)	4.36 ab (20.00)	3.36 bc (4.58)	2.38 c (−11.61)	2.28 bc (69.64)	.2010 b (28.56)	10.59 ef (3.16)	8.56 e (6.44)	4.36 c (11.22)
CRF17-18	12.96 cd (19.34)	7.29 f (-29.00)	6.13 d (−2.16)	5.80 bcd (54.91)	4.32 ab (19.01)	3.63 bc (12.89)	3.02 abc (12.50)	3.03 ab (125.07)	17.10 c (4.20)	14.42 cd (40.50)	9.26 de (15.19)	8.68 bc (121.30)
Mean	13.16	11.88	8.66	6.51	4.31	4.34	3.22	2.69	18.86	16.71	11.59	9.84
F-test	*	*	*	*	*	*	*	*	*	*	*	*
% CV	8.15	10.43	8.41	5.03	7.25	7.78	6.42	7.69	8.45	9.13	8.24	2.09
Rhizobacterial isolates (A)	**				**				**			
NaCl concentration (B)	**				**				**			
A × B	**				**				**			
LSD_(0.01)_ for (A × B)	1.5283				1.2928				2.1961			
% CV	9.35				21.84				30.21			

^1^ Controls = control-0, control-50, control-100, and control-150 at 0, 50, 100, and 150 mM NaCl, respectively; Mean (*n* = 3). Numbers in parentheses are percentage increases/decreases in shoot N, P, and K uptake in the KDML105 rice as compared to their respective controls. The average values followed by different letters within the same column were significantly different according to pairwise comparisons using an LSD test (*p* ≤ 0.01). *, ** Significant at the 0.05 and 0.01 probability level, respectively.

**Table 4:** Effect of ST-PGPR inoculation on calcium (Ca), magnesium (Mg), and sodium (Na) uptake by KDML105 rice seedlings under different NaCl concentrations.

ST-PGPR Strains	Ca				Mg				Na			
	(mg Ca plant^−1^)				(mg Mg plant^−1^)				(mg Na plant^−1^)			
	NaCl Concentrations (mM)				NaCl Concentrations (mM)				NaCl Concentrations (mM)			
	0	50	100	150	0	50	100	150	0	50	100	150
**Controls ^1^**	0.66 b (0.00)	0.49 bcd (0.00)	0.29 (0.00)	0.16 b (0.00)	1.68 b (0.00)	1.30 b (0.00)	0.79 cde (0.00)	0.48 c (0.00)	9.96 d (0.00)	10.45 cd (0.00)	9.38 de (0.00)	6.37 d (0.00)
**Organic rice farming**												
ORF4-13	0.67 b (1.82)	0.61 bcd (25.10)	0.44 (51.04)	0.30 ab (87.50)	1.38 b (−18.10)	1.82 b (40.74)	1.07 b (35.48)	0.73 abc (51.04)	10.21 cd (2.51)	13.45 b (28.72)	10.79 bcd (15.03)	9.45 b (48.35)
ORF10-12	0.93 b (40.61)	0.76 bc (56.38)	0.34 (16.67)	0.20 b (22.50)	1.57 b (−6.67)	1.72 b (32.71)	0.76 de (−4.55)	0.70 abc (45.83)	10.08 d (1.20)	14.24 b (36.28)	10.052 cd (7.16)	10.11 b (58.71)
ORF15-19	0.71 b (8.03)	0.54 bcd (11.93)	0.31 (6.25)	0.26 ab (63.13)	1.64 b (−2.20)	1.19 b (−8.18)	1.05 b (33.08)	1.01 ab (111.46)	9.95 d (−0.10)	12.04 bc (15.23)	12.27 ab (30.81)	10.88 b (70.80)
ORF15-20	0.84 b (27.27)	0.82 b (68.52)	0.58 (100.00)	0.46 a (184.38)	2.02 ab (20.00)	1.64 b (26.39)	1.55 a (95.45)	1.05 a (118.75)	13.48 b (35.34)	13.88 b (32.84)	13.54 a (44.35)	13.20 a (107.22)
ORF15-23	1.62 a (145.45)	1.98 a (306.58)	0.48 (66.67)	0.32 ab (102.50)	3.13 a (86.43)	3.95 a (204.94)	1.09 b (37.37)	1.05 a (119.38)	17.01 a (70.78)	16.30 a (56.00)	11.30 bc (20.47)	9.75 b (53.06)
**Conventional rice farming**												
CRF5-8	0.70 b (6.06)	0.42 d (−14.40)	0.30 (3.13)	0.26 b (40.63)	2.03 ab (20.83)	1.22 b (−5.71)	0.97 bc (22.73)	0.75 abc (56.25)	11.24 cd (12.85)	9.07 d (−13.20)	9.50 cde (1.28)	8.95 bc (40.50)
CRF14-15	1.02 ab (55.15)	0.46 cd (−5.56)	0.42 (45.83)	0.17 b (5.00)	1.73 b (2.86)	1.03 b (−20.83)	0.90 bcd (13.64)	1.01 ab (110.00)	10.56 cd (6.02)	9.50 cd (−9.08)	10.83 bcd (15.46)	10.42 b (63.58)
CRF16-3	0.79 b (20.00)	0.50 bcd (3.70)	0.22 (−23.61)	0.22 b (37.50)	1.69 b (0.71)	1.15 b (−11.42)	0.70 e (−11.11)	0.60 bc (25.00)	11.70 c (17.47)	10.22 cd (−2.19)	8.14 e (−13.22)	7.36 cd (15.54)
CRF17-18	0.83 b (25.45)	0.43 cd (−10.70)	0.22 (−25.00)	0.23 b (40.63)	1.73 b (2.86)	1.30 b (0.46)	1.05 b (32.95)	0.88 abc (82.29)	11.52 cd (15.66)	10.76 cd (2.98)	9.50 cde (1.28)	9.03 bc (41.76)
Mean	0.88	0.70	0.36	0.25	1.86	1.63	0.99	0.83	11.57	12.99	10.53	9.55
F-test	*	*	ns	*	*	*	*	*	*	*	*	*
% CV	3.34	2.94	5.23	3.84	8.53	3.85	8.33	2.18	5.88	8.48	7.77	9.08
Rhizobacterial isolates (A)	**				**				**			
NaCl concentration (B)	**				**				**			
A × B	**				**				**			
LSD_(0.01)_ for (A × B)	0.2823				0.5456				1.3728			
% CV	31.73				25.28				7.57			

^1^ Controls = control-0, control-50, control-100, and control-150 at 0, 50, 100, and 150 mM NaCl, respectively; Mean (*n* = 3). Numbers in parentheses are percentage increases/decreases in shoot Ca, Mg, and Na uptake in the KDML105 rice as compared to their respective controls. The average values followed by different letters within the same column were significantly different according to pairwise comparisons using the LSD test (*p* ≤ 0.01). *, ** Significant at the 0.05 and 0.01 probability level, respectively.

**Table 5:** Effects of ST-PGPR inoculation on K^+^/Na^+^ ratio in KDML105 rice seedlings under different NaCl concentrations.

ST-PGPR Strains	K^+^/Na^+^ Ratio			
	NaCl Concentrations (mM)			
	0	50	100	150
**Controls ^1^**	1.65 ab	0.99 c	0.86 b	0.62 ef
**Organic rice farming**				
ORF4-13	1.67 ab	1.34 ab	1.03 ab	1.01 cd
ORF10-12	1.72 ab	1.04 c	0.95 b	1.07 c
ORF15-19	1.69 ab	1.51 a	1.35 a	1.45 b
ORF15-20	1.33 b	1.17 bc	1.09 ab	0.69 def
ORF15-23	1.66 ab	2.34 a	1.49 ab	0.91 cde
**Conventional rice farming**				
CRF5-8	1.61 ab	1.37 ab	1.30 a	1.06 a
CRF14-15	1.75 a	1.45 a	1.17 ab	1.06 c
CRF16-3	1.80 a	1.04 c	1.05 ab	0.59 f
CRF17-18	1.48 ab	1.34 ab	0.98 b	0.96 cd
Mean	1.64	1.27	1.10	0.94
F-test	*	*	*	*
% CV	10.60	10.32	13.366	13.75
Rhizobacterial isolates (A)	**			
NaCl concentration (B)	**			
A × B	**			
LSD_(0.01)_ for (A × B)	4.81			
% CV	11.84			

^1^ Controls = control-0, control-50, control-100, and control-150 at 0, 50, 100, and 150 mM NaCl, respectively. The average values followed by different letters within the same column were significantly different according to all pairwise comparisons using the LSD test (*p* ≤ 0.01). *, ** Significant at the 0.05 and 0.01 probability level, respectively.

**Table 6:** Effects of the inoculation of rhizobacterial isolates from organic and conventional farming practices on the IAA production and 2AP level of KDML105 rice seedlings and rhizobacterial count under different salt stress conditions.

Strain	Genus	IAA Production (µg IAA mL^−1^)	2AP Level of KDML105 Rice Seedlings (μg·kg^−1^)				Rhizobacterial Population (CFU mL^−1^)			
			NaCl (% *w*/*v*)				NaCl (% *w*/*v*)			
			0	50	100	150	0	1	2	3
**Organic farming ^2^**										
ORF4-13	*Sinomonas* sp.	155.1	11.01	13.23	7.55	4.87	8.7 × 10^8^	2.3 × 10^8^	8.3 × 10^7^	6.7 × 10^7^
ORF10-12	*Enterobacter* sp.	47.7	14.31	18.7	12.87	7.14	2.2 × 10^9^	2.7 × 10^8^	1.0 × 10^8^	1.7 × 10^7^
ORF15-19	*Micrococcus* sp.	147.2	14.64	18.71	8.54	6.53	2.3 × 10^9^	1.1 × 10^9^	8.3 × 10^8^	1.5 × 10^8^
ORF15-20	*Micrococcus* sp.	127.8	15.39	18.24	6.62	5.88	7.2 × 10^8^	1.5 × 10^8^	1.3 × 10^7^	1.2 × 10^7^
ORF15-23	*Sinomonas* sp.	155.6	15.64	19.61	10.13	6.22	2.1 × 10^9^	1.3 × 10^9^	8.3 × 10^8^	2.1 × 10^8^
**Conventional farming ^2^**										
CRF5-8	unidentified	ND ^1^	12.44	13.64	8.21	4.65	1.2 × 10^9^	2.5 × 10^8^	6.2 × 10^7^	3.5 × 10^7^
CRF14-15	*Sinomonas* sp.	84.5	10.65	11.58	6.92	4.41	9.7 × 10^8^	3.3 × 10^8^	2.7 × 10^8^	6.7 × 10^7^
CRF16-3	*Burkholderia* sp.	7.3	14.06	17.43	10.12	9.43	3.8 × 10^6^	6.7 × 10^5^	3.3 × 10^5^	1.7 × 10^5^
CRF17-18	*Bacillus* sp.	55.1	11.03	12.01	6.43	5.75	1.1 × 10^9^	2.5 × 10^8^	7.8 × 10^7^	3.5 × 10^7^

^1^ ND = not detectable; ^2^ farming practice. Adapted from Chinachanta and Shutsrirung [7].

## Data Availability

Data sharing does not apply to this article as no datasets were generated or analyzed during the current study.
